# Crossed aphasia in a left-handed patient with non-fluent variant of primary progressive aphasia with left asymmetric brain SPECT

**DOI:** 10.1590/1980-5764-DN-2022-0095

**Published:** 2023-12-11

**Authors:** Paulo Roberto de Brito-Marques, Janaina Mariana de Araujo Miranda Brito-Marques

**Affiliations:** 1 Universidade de Pernambuco, Faculdade de Ciências Médicas, Unidade de Neurologia, Unidade de Cognição e Neurologia Comportamental, Recife PE, Brazil.; 2 Universidade de Pernambuco, Hospital Universitário Oswaldo Cruz, Recife PE, Brazil.

**Keywords:** Tomography, Emission-Computed, Single-Photon, Neurodegenerative Diseases, Dominance, Cerebral, Dementia, Aphasia, Primary Progressive, Tomografia Computadorizada de Emissão de Fóton Único, Doenças Neurodegenerativas, Dominância Cerebral, Demência, Afasia Primária Progressiva

## Abstract

Primary progressive aphasia is a clinical syndrome caused by neurodegeneration of areas and neural networks involved in language, usually in the left hemisphere. The term “crossed aphasia” denotes an acquired language dysfunction caused by a lesion in the ipsilateral hemisphere to the dominant hand.

**Objective:**

To describe a case of crossed aphasia in a 60-year-old left-handed patient with a non-fluent variant of primary progressive aphasia diagnosis (age of onset=52), evidenced by a left asymmetry on brain SPECT scan.

**Methods:**

Clinical and family history, the Edinburgh Handedness Inventory, Measurement of Functional Activities in Older Adults in the Community, the “Mini-Mental State Examination”, the Trail Making Test, the Tower of London, and the Neuropsychological assessment for dementia, and neuroimaging studies were carried out.

**Results:**

Neuropsychological assessment showed severe cognitive impairment, especially regarding language. The magnetic resonance imaging showed important signs of cortico-subcortical atrophy, with predominance in the frontal and temporal lobes. The single-photon emission computed tomography scan showed moderate to severe hypoperfusion in the left cerebral hemisphere, including the hippocampus.

**Conclusion:**

We described a clinical case of crossed aphasia in a left-handed woman with a non-fluent variant of primary progressive aphasia with asymmetry on brain SPECT, mainly on the left, followed up for seven years.

## RESUMO

A afasia progressiva primária é uma síndrome clínica causada por uma neurodegeneração de áreas e redes neurais envolvidas na linguagem, geralmente no hemisfério esquerdo. O termo “afasia cruzada” denota uma disfunção adquirida de linguagem causada por uma lesão no hemisfério ipsilateral da mão dominante.

**Objetivo:**

Relatamos um caso de afasia cruzada em uma paciente de 60 anos, canhota, com um quadro clínico de afasia progressiva primária variante não fluente (idade de início=52), evidenciada por assimetria no SPECT cerebral à esquerda.

**Métodos:**

Foram realizados para o diagnóstico do caso: história clínica e familiar, o Inventário de Dominância de Edinburgh, a Escala de Atividades Funcionais de Pfeffer, o Miniexame do Estado Mental, o Teste das Trilhas, o Teste da Torre de Londres, a Avaliação Neuropsicológica Adequada às Demências e exames de neuroimagem.

**Resultados:**

A avaliação neuropsicológica mostrou comprometimento cognitivo severo, principalmente sobre a linguagem; a ressonância magnética do crânio mostrou sinais de involução córtico-subcortical, com predominância nos lobos frontal e temporal e a cintilografia cerebral por emissão de fóton único mostrou hipoperfusão moderada a severa no hemisfério cerebral esquerdo, incluindo o hipocampo.

**Conclusão:**

Registramos um caso clínico de afasia cruzada em uma paciente canhota com afasia progressiva primária variante não fluente com assimetria no SPECT cerebral, principalmente à esquerda, seguida há sete anos.

## INTRODUCTION

Primary progressive aphasia is a clinical syndrome caused by a neurodegeneration of areas and neural networks involved in language, usually in the left hemisphere. The term “crossed aphasia” denotes an acquired language dysfunction caused by a lesion in the ipsilateral hemisphere to the dominant hand. Non-fluent/agrammatic variant of primary progressive aphasia (nfvPPA) is characterized by non-fluent, labored speech and agrammatism with little or no comprehension disabilities. On brain imaging, atrophy and hypometabolism are usually seen in the left inferior frontal gyrus and anterior insula. Pathologically, nfvPPA is most likely regarded as tauopathy^1^. Crossed aphasia has been reported mainly as post-stroke aphasia resulting from brain damage ipsilateral to the dominant right hand^2^, and accounts for about 3% of aphasia cases of vascular cause^3^. However, few cases of primary progressive crossed aphasia have been reported^4-7^. There is a prevalence of left-handedness ranging between 6 and 12 % in Germany; as a consequence, left-handed patients with nfvPPA are very rare^4^. In 2002, Drzezga’s group first described a pattern of right hemispheric hypometabolism using 18-fluorodeoxyglucose (18F-FDG) positron emission tomography (PET) in 2 left-handed patients with nfvPPA^4^. In 2007, Repetto et al. described right frontal hypoperfusion in a right-handed patient with nfvPPA, using single photon emission computed tomography (SPECT) scan^5^, and the groups of Demirtas-Tatlidede in 2012 and Parente in 2015 published cases of crossed logopenic aphasia in right-handed patients, demonstrated with SPECT scan and PET/SCAN, respectively^6,7^. However, there are relationships between degenerative and vascular aphasias. Semantic variant PPA and Wernicke aphasia are characterized by fluent speech with naming and comprehension difficulty; these syndromes are associated with diseases in different portions of the left temporal lobe. Patients with non-fluent/agrammatic variant PPA or Broca aphasia have non-fluent speech with grammatical difficulty; these syndromes are associated with diseases centered in the left inferior frontal lobe. Patients with logopenic variant PPA or conduction aphasia have difficulty with repetition and word finding in conversational speech; these syndromes are associated with disease in the left inferior parietal lobe. While PPA and stroke aphasias resemble one another, this article also presents their distinguishing features^8^. Here, you will find a clinical case of crossed nfvPPA in a left-handed woman confirmed by clinical and family history, neuropsychological assessment, SPECT scan, and follow-up during 7 years after diagnosis.

## METHODS

A clinical case of crossed aphasia in a left-handed patient with nfvPPA was diagnosed, according to Gorno-Tempini et al.^1^. Clinical and family history, the Edinburgh Handedness Inventory^9^, Measurement of Functional Activities in Older Adults in the Community^10^, the Mini-Mental State Examination (MMSE)^11^, the Trail Making Test^12^, the Tower of London^13^, the Neuropsychological assessment appropriate for dementias^14^, magnetic resonance imaging (MRI), SPECT scan were carried out. The patient was also submitted to other examinations such as blood screening for dementia and did not undergo cerebrospinal fluid (CSF) biomarkers and PET/SCAN. She was examined every four to six months, except during the pandemic. Speech therapy was assessed in three consecutive weekly sessions and was monitored for six months.

The authors declare that they have followed the protocols of their work center on publishing patient data. Consent for publication was obtained. The study received the approval of the local Ethics Committee.

## CASE REPORT

A 60-year-old monolingual woman, single, an accountant with 11 years of education came to receive medical care attention at the cognitive and behavioral neurology outpatient clinic at Universidade de Pernambuco on July 7^th^, 2015. Eight years ago, the patient had three episodes of syncope for one year, each followed by transient stuttering without clinical investigation. According to the sister, after one year, the patient changed the names of family members of the same sex without realizing it; she had difficulty finding words during a conversation; she could not form a sentence nor read, write, or make house money calculations; she was unable to express herself and say what she was thinking, words did not come to mind: verbal fluency decreased progressively and she occasionally stuttered at the beginning of a sentence. She had a family history of dementia and Down syndrome. The subject had controlled glaucoma, but had no language impairment or other clinical illnesses, such as seizures.

Neurological examination was normal except for the appearance of a Myerson’s sign. Patient’s left-handedness was confirmed by the Edinburgh handedness inventory^9^. Furthermore, the patient and her sisters reported that she was strongly left-handed since her first limb activities at childhood and had 3 left-handers in their family history. Upon admission, she presented insight and independence in self-care and housekeeping, with mild impairment (9/30), confirmed by Pfeffer’s scale^10^, but also showed moderate emotional detachment from behavior and apathy. On detailed examination of language, speech was characterized by spontaneous muteness, but the patient repeated syllables and spoke simple compound words and compound sentences. Agrammatism was evident in speech. Despite progressive speech disturbances, the semantic domain was mildly impaired and her comprehension was preserved, but her repetition was of isolated words. The patient would not read nor write. On the other hand, the general neuropsychological evaluation showed deficits in attention, visuospatial and executive functions, visual memory, and praxis. Difficulties in expressing language made her somewhat distressed and hasty, but no behavioral disturbances were reported. The patient’s cognitive performance is shown in Table 1 ^10-15^. She scored 03/30 on MMSE^11^ in the Pierre Marie subtest. There were no abnormalities in routine blood tests, including thyroid function testing, vitamin B12, and syphilis screening.

**Table 1.** Neuropsychological data collected from our patient.

Neuropsychological data		Row score
Mini-Mental State Examination^11^		03
Pfeffer’s functional activities scale^10^		9/30
Attention and executive functions^12,13^	Trail Making Test – part A	00
	Trail Making Test – part B	00
	London tour	00
Memory^14,15^	Visual reproduction of the three cards	0/4
	Mesulam casual memory copy	2/6
	Mesulam casual memory recall	0/6
Language^15^	Denomination – figures	2/30
	Phonological fluency – P	2/18
	Phonological semantic – animals	1/18
	Oral comprehension	15/19
	Written words	0/12
	Reading words aloud	2/7
	Repetition of words	8/15
	Calculations – subtractions	0/5
Visuo-spatial abilities^15^	Monomanual pantomimes	4/5
	Bimanual pantomimes	2/5
	Monomanual ideomotor apraxia	0/5
	Bimanual ideomotor apraxia	3/5
	Constructive apraxia	0/4

**Figure 1.**
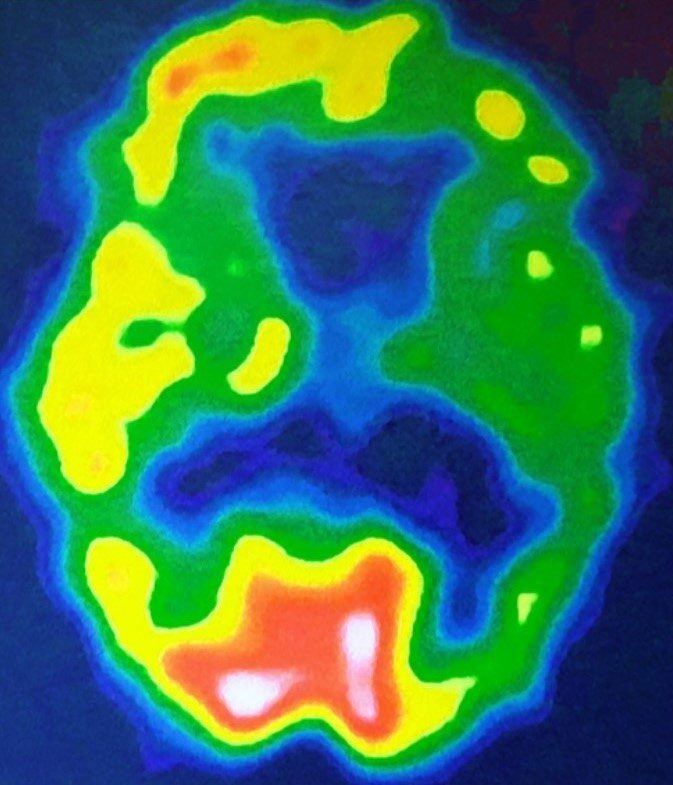
Axial SPECT scan demonstrated severe hypometabolism in the left hemisphere in the fronto-parieto-temporal cortex, including hypometabolism in the left striatum and thalamus.

MRI showed signs of cortico-subcortical involution characterized by enlargement and grooves, Sylvian fissures and basal cistern, beyond what was expected for the age range, with mild to moderate predominance in the frontal and left temporal lobes. There is prominence of the choroidal fissures and temporal horns of the lateral ventricles, including the hippocampus, but due to wasting of the neocortex. Compensatory dilation of the lateral ventricles. Fazekas’s scale = 2. SPECT scan showed moderate to severe hypoperfusion in the left cerebral hemisphere, including the hippocampus (Figure 1).

### Follow-up

**Figure 2.**
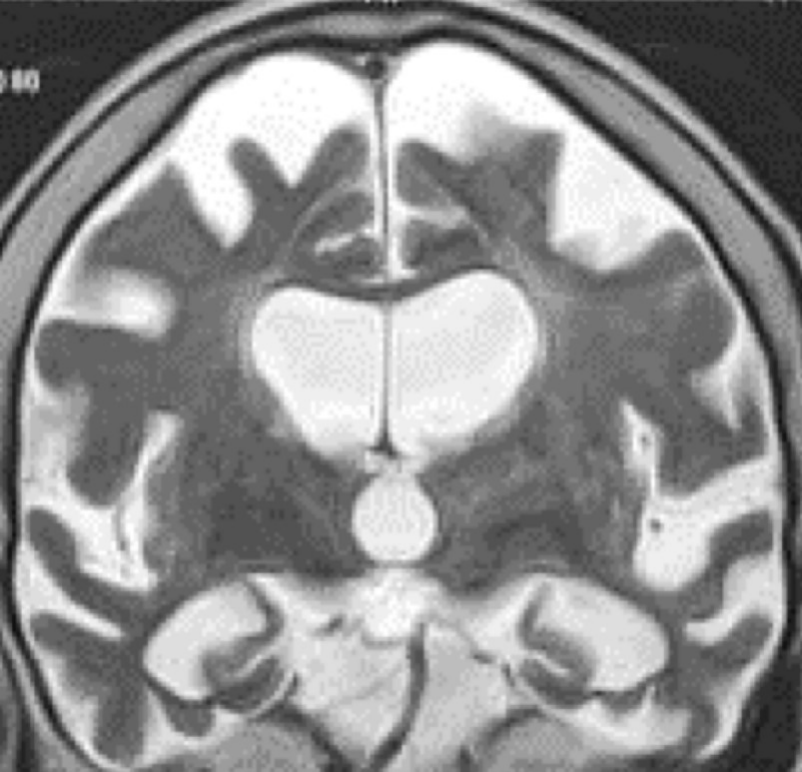
Coronal magnetic resonance imaging demonstrated significant asymmetric diffuse cortical atrophy, especially in the frontotemporal lobes, being slightly more to the left.

Four years after the diagnosis of crossed nfvPPA, her MRI showed important diffuse asymmetric cortical atrophy, especially in the frontotemporal lobes, being slightly more to the left (Figure 2).

**Figure 3.**
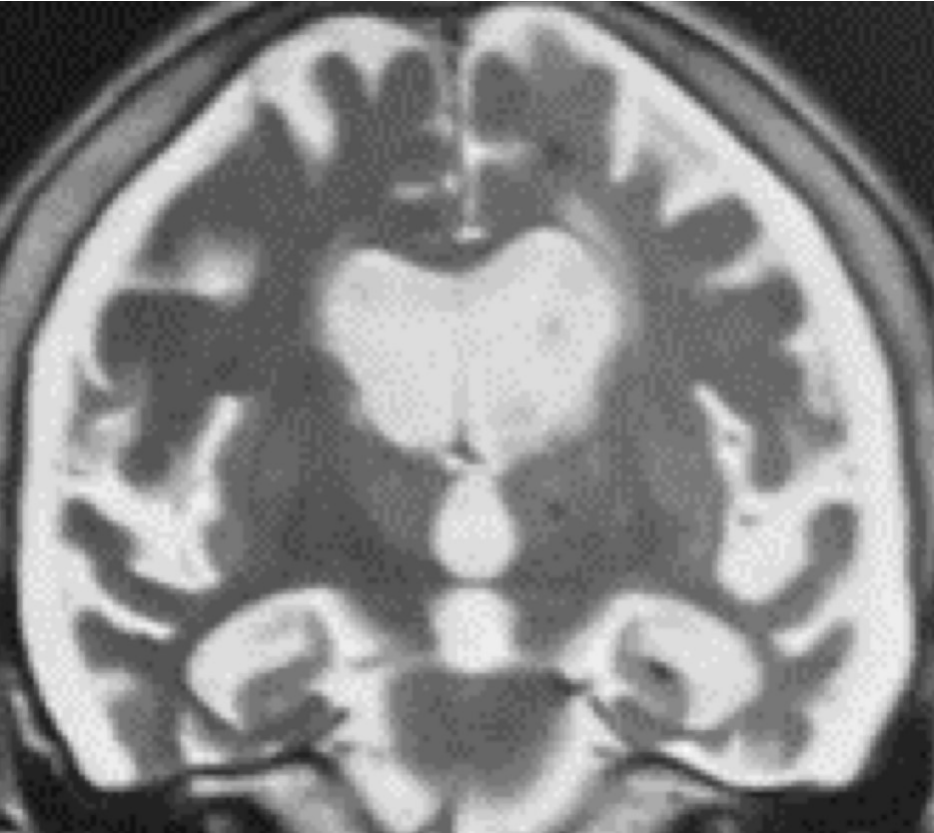
Coronal magnetic resonance imaging demonstrated diffuse cerebral atrophy slightly more prominent in the left hemisphere, the hippocampi showed a 3/4 Scheltens’ scale and extensive bilateral hemorrhagic subdural hematoma.

After another 3 years of follow-up, the patient became mute and did not recognize her family. She has dementia confirmed by Pfeffer’s scale (30/30), and MMSE screening test (00/30), although she walked without assistance inside her home. Sever years after diagnosis, the patient got up in the morning and fell to the ground having a seizure. On the same day, CT examination showed no new changes. Her MRI showed diffuse cerebral atrophy slightly more prominent in the left hemisphere, the hippocampi showed a 3/4 Scheltens’ scale and extensive bilateral hemorrhagic subdural hematoma (Figure 3), but treatment was conservative. The patient lacks autonomy and independence in basic activities of daily living.

## RESULTS

Neuropsychological assessment showed severe cognitive impairment, especially in language. MRI showed important signs of corticosubcortical atrophy, predominantly in the frontal and temporal lobes. SPECT scan showed moderate to severe hypoperfusion in the left cerebral hemisphere, including the hippocampus (Table 1).

## DISCUSSION

To our knowledge, this report documents for the first time the occurrence of an association between a case showing clinical findings compatible with nfvPPA and imaging abnormalities in the left hemisphere, indicating left hemisphere dominance for language in a left-handed patient. Up to now, few cases of crossed neurodegenerative aphasia have been reported^6,7^ presenting mainly as nfvPPA subtype^5^. It could be a case of crossed nfvPPA, but this cannot be said for sure, since the patient is left-handed and there is a significant percentage of left-handers with left-brain dominance. According to the neuropsychological data collected from our patient (Table 1), it could be a severe case of crossed nfvPPA, but as the patient’s evolution was not monitored from the onset of symptoms, it is not possible to say with certainty. These crossed aphasia syndromes have been described mainly in post-stroke^16^ and any cases of PPA in right-handed people^5,6,17^. However, primary progressive and stroke aphasia syndromes disrupt the left perisylvian language network, resulting in identifiable degenerative and vascular aphasic syndromes^8^. Reliable clinical, neuropsychological, and neuroimaging data were identified to diagnose our left-handed patient with crossed nfvPPA with moderate hypoperfusion in the left hemisphere. This term denotes any aphasic syndrome resulting from brain damage ipsilateral to the dominant left hand, and with a lower percentage in the non-dominant cerebral hemisphere. According to brain dominance, about 95% of the population are right-handed, and 4% are left-handed. Of this 4%, 70% have left brain hemisphere dominance, 15% have dominance in the right hemisphere, and another 15% have dominance in both hemispheres^18^. Our left-handed patient presented clinical disturbances in language for the left cerebral hemisphere ipsilateral dominant hand, evidenced by asymmetry on SPECT before MRI. On the other hand, a multimodal neuroimaging study was performed in a patient with nfvPPA, with frontoparietal involvement to the right, which showed that the presence of aphasia, despite right hemispheric involvement, was due to premorbid bilateral lateralization of language, evidenced by functional MRI^2^. In 2002, Drzezga et al.^4^ reported two left-handed subjects with typical symptoms of nfvPPA, whose 18F-Fluordeoxiglicose (18FFDG) PET revealed an asymmetric right-hemispheric pattern of reduced glucose metabolism in the frontal and temporoparietal cortex. These findings support the hypothesis that PPA can be considered as a complex of symptoms rather than a disease entity. For instance, Mesulam et al.^19^ studied 58 autopsies of patients with APP and showed the distribution of nfvAPP biomarkers with 65% of tau protein. Following the same principle, Brito-Marques et al.^20^ published a case of corticobasal degeneration at autopsy with tauopathy. In this case, despite the long duration of the disease and cerebral atrophy (8 years before diagnosis), there was an asymmetric cerebral SPECT that showed a slight contribution from the right hemisphere to language, as was described^21-23^.

In conclusion, we reported a case of crossed nfvPPA in a left-handed woman with a 15-year clinical history of a rare clinical case. Neuroimaging techniques used in crossed nfvPPA, such as SPECT scan, showed asymmetric brain hypoperfusion long before asymmetry on the MRI. Can we speculate that the expected language changes do not appear early with the same evolution when premorbid lateralization of language occurs in left-handers? In addition, these findings support that primary progressive aphasia should be considered as a syndrome characterized by asymmetric neurodegeneration of brain regions and neural networks involved in language. Most left-handed patients have less pronounced language dominance compared to right-handed ones. Left-handed patients, however, do not represent a uniform group and some significant differences in language disorders are found, depending on which hand is used for writing^24^. Further analysis of left-handed individuals with left-hemisphere language dominance is needed to enhance our understanding of the role of cerebral language dominance of left-handed individuals.

The most important limitation of our study was the patient’s delay in seeking the neurological service when other cognitive functions were already impaired, exceeding the 2-year period expected for language^15^, which is not an uncommon fact in the Public Service in cases of nfvPPA^25^. On the other hand, the degree of anomia and spontaneous speech were extremely impaired, expressing a severe degree of aphasia, but the functional activities of daily living were acceptable^10^. The clinical history of nfvPPA was conclusive with a familial turning point that helped formulate a clinical rationale. As the patient’s evolution was not followed-up from the onset of symptoms, it is not possible to say with certainty that this is a case of crossed nfvPPA, although it is suggestive.
